# The Role of Cohesion Policy Funds in Decreasing the Health Gaps Measured by the EURO-HEALTHY Population Health Index

**DOI:** 10.3390/ijerph17051567

**Published:** 2020-02-29

**Authors:** Paula Santana, Ângela Freitas, Cláudia Costa, Iwa Stefanik, Gonçalo Santinha, Thomas Krafft, Eva Pilot

**Affiliations:** 1 Department of Geography and Tourism, Faculty of Arts and Humanities, University of Coimbra, 3004-530 Coimbra, Portugal; 2 Centre of Studies in Geography and Spatial Planning (CEGOT), University of Coimbra, 3004-530 Coimbra, Portugal; angela.freitas@uc.pt (Â.F.); claudiampcosta@uc.pt (C.C.); iwastefanik@gmail.com (I.S.); g.santinha@ua.pt (G.S.); 3 Department of Social, Political and Territorial Sciences; Governance, Competitiveness and Public Policies Research Unit (GOVCOPP), University of Aveiro, 3810-193 Aveiro, Portugal; 4 Department of Health, Ethics and Society, Faculty of Health, Medicine and Society, Maastricht University, P.O. Box 616, 6200 MD Maastricht, The Netherlands; thomas.krafft@maastrichtuniversity.nl (T.K.); eva.pilot@maastrichtuniversity.nl (E.P.); 5 Maastricht Centre for Global Health, Maastricht University, P.O. Box 616, 6200 MD Maastricht, The Netherlands

**Keywords:** European Union, regional health inequalities, cohesion policy funds, determinants of health, population health index, less developed regions, intervention field

## Abstract

Social, economic, and environmental differences across the European Union significantly affect opportunities to move forward in achieving greater equity in health. Cohesion Policy (CP) funds can contribute positively through investments in the main determinants of health. The aim of this study is to analyze to what extent the planned investments for 2014–2020 are addressing the regional health gaps, in light of the population health index (PHI), a multidimensional measure developed by the EURO-HEALTHY project. The operational programs of all regions were analyzed, namely, the CP planned investments by field of intervention. Analysis of variance was performed to examine whether the regional scores in the PHI dimensions were statistically different across regions with different levels of development (measured by gross domestic product (GDP)). Results show that 98% of regions with worse performances on the PHI are less developed regions. Overall, all regions present planned investments in intervention fields linked to dimensions appraised within the PHI (e.g., employment, income, education, pollution). Yet, more needs to be done to focus regional investments in health determinants where regions still lag behind. The PHI has the potential to inform future CP restructuring, providing evidence to extend the current eligibility criteria to other dimensions beyond the GDP.

## 1. Introduction

The factors that are causing differences in the social, economic, and environmental conditions in the European Union (EU) are also leading to health inequalities that, in turn, undermine efforts to achieve effective social and spatial cohesion [1,2,3,4]. Health became an evolving concept since the landmark World Health Organization (WHO) definition in 1948, which stated that “health is a state of complete physical, mental, and social well-being and not merely the absence of disease or infirmity”. Concurrent to the discussion of the broader and holistic scope of health is the recognition that health inequities are shaped by structural and systematic differences in the social, economic, and environmental determinants of health. These are the conditions in which people are born, grow, live, learn, work, play, love, and age, which affect a wide range of health, functioning, and quality-of-life outcomes, including education, income, employment, housing, the physical environment, public safety, the social environment, transportation, etc. [5,6,7]. The interconnectedness of health with all policies acting on its determinants [8], originally stated in the Alma Ata Declaration on Primary Health Care [9] and the Ottawa Charter for Health Promotion [10], introduced a new spectrum for looking at health and implied a need for an integrated approach to population health [11,12].

For the purpose of this article, population health is understood as described and popularized by Kindig and Stoddart [13], which considers the “health outcomes and their distribution within a population, the patterns of determinants that influence such outcomes, and the policies that influence the optimal balance of determinants” [13]. The underlying assumption focuses on improving the health of the population rather than individuals and on promoting health equity through actions that target their main drivers, which are the social, economic and environmental conditions. Regarding health equity, it is referred here as “the principle underlying a commitment to reduce and, ultimately, eliminate disparities in health and in its determinants” [14].

### 1.1. The EU Cohesion Policy and Health

The concern for greater cohesion and solidarity—the key common principles of the EU—remains an essential feature underlying the process of integration and the creation of a “Europe of Regions” [15]. Since 1988, the EU invests in a regional policy to provide strategic assistance for European regions to achieve their full economic potential and development [16]. Measures taken toward this stated objective are implemented through EU regional and urban development policy, known as Cohesion Policy (CP) [17]. CP is the EU’s strategy to stimulate, strengthen and support consistent development of its Member States (MS) and regions by reducing the existing economic, social, and territorial inequalities amongst them [18] through the allocation of funds in disadvantaged areas and sectors (Article 174 of the Treaty on the Functioning of the EU). This unique financial instrument, one which follows a multi-level governance model involving national, regional, and local authorities [19], is operationalized through the operational programs (OP), a multi-fund program, bringing together investments from the European Regional Development Fund (ERDF), the European Social Fund (ESF), and the Cohesion Fund [20]. The first contributes to economic and social cohesion in the EU by reducing imbalances amongst its regions and by enhancing competitiveness and territorial cooperation. The second invests in people by promoting employment, social inclusion, and education opportunities. The third provides support exclusively for the poorer regions of Europe.

The CP budget accounts for approximately one-third of the entire EU budget (€351.8 billion in the period 2014–2020) [21], constituting significant opportunities for health-related investments [16], particularly toward the reduction of inequalities in key determinants of health [22]. There is extensive evidence that low socioeconomic status and low income are associated with poor health outcomes, particularly with respect to high mortality [2,23,24,25,26]. By allocating funds to the regions where development is lagging behind, CP could act as a major driver of health equity [27,28,29]. Most of the funds are targeted to those very regions with a gross domestic product (GDP) per capita under 75% of the EU average. This is the basis for the categorization of a region and its eligibility to receive funds. Recently, there was some debate around the use of the GDP as a single indicator to measure regional development. Beyond the different levels of GDP, there is an unequal distribution and allocation of other resources, power, and access to services, thus creating inequalities between regions and countries, which in turn generates inequities in health. CP contributes to reducing the gap between the better and worse performing regions by delivering funds that support job creation, business competitiveness, economic growth, sustainable development, and improve citizens’ overall quality of life. These domains of intervention address conditions and factors influencing health positively or negatively—the so-called social, economic, environmental, and lifestyle-related determinants of health, i.e., factors that can be influenced by political, commercial and individual decisions—as opposed to age, sex, and genetic factors, which also influence health but are not, on the whole, open to influence by political or other types of policy [30,31].

Furthermore, CP funds help to deliver many EU objectives, as it complements policies from different sectors, such as those dealing with education, employment, energy, and the environment. Article 168 of the Treaty on the Functioning of the EU provides a scope for the “mainstreaming” of health into other policy areas, which required all European Commission (EC) activities to take health into consideration [32]. This is in line with the Health in All Policies (HiAP) framework, which was first introduced in Europe during the 2006 Finnish presidency of the EU. This framework aims to boost collaboration on the basis of greater inclusion of health considerations in policymaking across those policy sectors that influence health, such as environment, transportation, agriculture, land use, housing, and education, among others [33]. HiAP is currently a central element in the recent EU Health Strategy and also a part of the CP. However, the actual effectiveness of its official integration and impact assessment seems to be low, at both the EU and the country levels [32].

### 1.2. The EURO-HEALTHY Population Health Index

The Horizon 2020 EU-funded project EURO-HEALTHY (shaping EUROpean policies to promote HEALTH equitY), among other projects, looked to CP funds as resources with significant potential to decrease the health gap between the EU regions [4,34]. The project aimed to advance knowledge regarding population health across Europe, with particular attention at NUTS (Nomenclature of Territorial Units for Statistics) level 2 regions, the geographical and administrative level corresponding to the category of eligibility for receiving CP funds.

Due to the comprehensive nature of health inequalities, including, among others, the various social, cultural, behavioral, environmental, and political differences amongst the respective healthcare systems across Europe [4,35], there is a need for enhanced evidence on the main drivers of regional health inequality to better guide policies and the allocation of CP funds.

Aligned with this, EURO-HEALTHY constructed a population health index (PHI)—a multidimensional measure—to evaluate population health inequalities across 10 areas of concern, with 17 dimensions and 39 indicators of health determinants and health outcomes across 269 NUTS 2 regions [4].

The construction of this index relied on the following key assumptions: (i) it is informed not only by the current state of the art and evidence on the different dimensions that population health entails, but also by the way health experts and stakeholders interpret that evidence and make use of their knowledge to evaluate population health [36]; (ii) it uses a sound methodology (MACBETH—measuring attractiveness by a categorical-based evaluation technique) and makes use of principles and concepts of multicriteria value measurement that avoid the common critical mistakes that often occur in the construction of indices [36,37]; (iii) it enables aggregate and disaggregated analyses of multilevel indices (e.g., for health determinants vs. outcomes, for areas of concern and for dimensions) [37].

Founded on these assumptions, the index presents a bottom-up hierarchical structure, providing an evidence-based analysis of regional health gaps in two indices—health determinants and health outcomes—divided into sub-indices corresponding to areas of concern that reflect broad domains to analyze population health: (i) economic conditions, social protection, and security, (ii) education, (iii) demographic change, (iv) lifestyle and health behaviors, (v) physical environment, (vi) built environment, (vii) road safety, (viii) healthcare resources and expenditure, (ix) healthcare performance, and (x) health outcomes. Each area of concern is further divided into dimensions, which are independent evaluation axes for appraising population health, and are made operational by one or more indicators [4]. Indicators were selected through a wide Web-Delphi process where an international panel of 80 stakeholders and experts from multidisciplinary fields shared their views on which factors were deemed relevant to evaluate European population health [38]. The indicator data were collected for the year 2014 and retrieved from different official bodies, such as EUROSTAT, the European Environment Agency (EEA), and the World Health Organization (WHO) [39].

The results of its application to all NUTS 2 regions are publicly accessible from the WebGIS healthyregionseurope (https://healthyregionseurope.uc.pt) and are published in the “Atlas of Population Health in European Union Regions” [4].

This paper aims to analyze the regional planned investments for allocating CP funds in the period 2014–2020 and discuss their potential to reduce regional health inequalities, in light of the evidence provided by the EURO-HEALTHY PHI. Our underlying research questions are as follows:

(1) Do the intervention fields with planned allocation of CP funds match the dimensions where regions are lagging behind, as identified in the population health index?
(2) Are there any categories/intervention fields that are relevant for closing regional health gaps but appear to be currently underrepresented in CP planned investments?

## 2. Materials and Methods

### 2.1. Materials

This study is based on two main data sources: (i) the EURO-HEALTHY population health index and (ii) operational programs (OP) for Cohesion Policy Funding 2014–2020, involving the areas of intervention with planned allocation of funds in each region. All EU countries and regions were included in the analysis (269 NUTS 2), except for the seven outermost regions and autonomous cities due to data availability.

**Figure 1:**
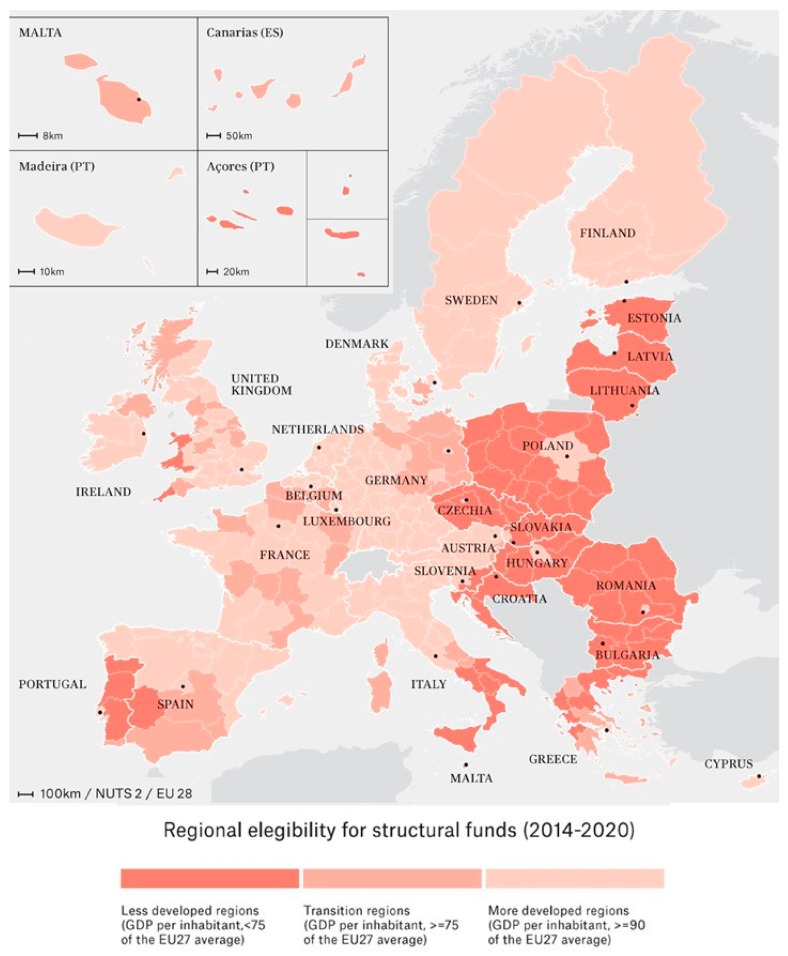
Regional eligibility map, 2014–2020.

All the analyses conducted in this study take into account the three categories of regional eligibility for receiving CP funds, based on the regional GDP per capita: (1) less developed regions (LD) (GDP <75% of the EU-27 average), (2) transition regions (TR) (GDP between 75% and 90% of the EU-27 average), and (3) more developed regions (MD) (GDP >90% of the EU-27 average) (Figure 1).

#### 2.1.1. Population Health Index

**Figure 2:**
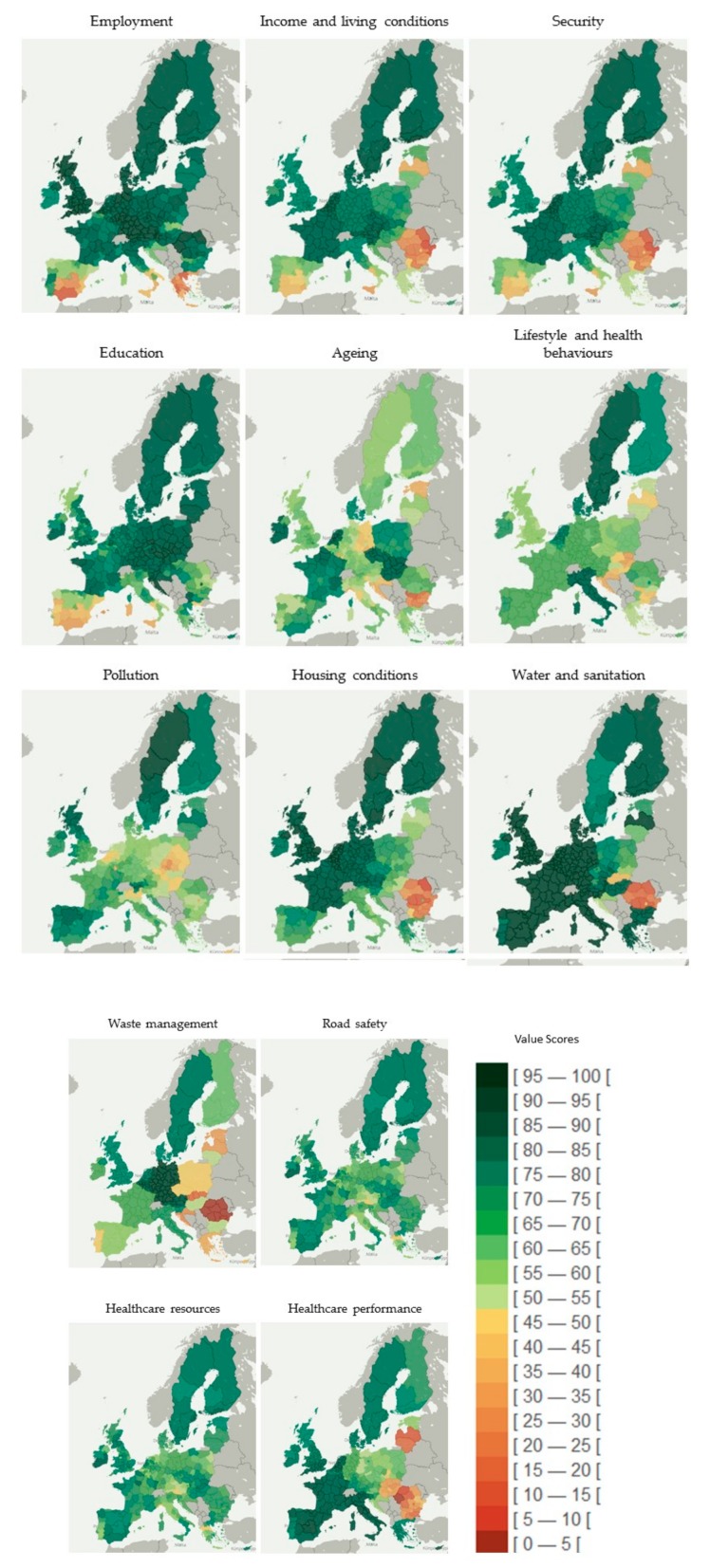
Geographical distribution of the population health index at regional level and across dimensions of health determinants. Note: The regional value-scores are classified in an equal interval scheme. This method divides the values into equal size range considering the minimum and maximum scores (from 0 to 100). The color coding of classes uses a gradation inspired by a traffic light system; red represents worse population health and green represents better health.

For this study, we used the value scores presented by all EU regions on the following dimensions of the health determinants: (i) employment; (ii) income and living conditions; (iii) security; (iv) education; (v) aging; (vi) lifestyle and health behaviors; (vii) pollution; (viii) housing conditions; (ix) water and sanitation; (x) waste management; (xi) road safety; (xii) healthcare resources; (xiii) healthcare performance (Figure 2). These dimensions correspond to policy intervention areas with impact on population health (positive or negative) and are associated to one or more determinants of health (Table 1). The value scores range between 0 (worse health) and 100 (better health) and were obtained through the application of a socio-technical methodological approach for the construction of indices, combining the multicriteria method MACBETH with Web-Delphi and decision conferencing processes [4,34,36,37]. These processes, using intuitive protocols of questioning, informed the shape of value functions (added value of improvements in indicators) and the weighting coefficients (importance of closing gaps in indicators). More information on the methodology and on the hierarchical multicriteria model can be found in Bana e Costa et al. [36,37].

#### 2.1.2. Cohesion Policy Funding for 2014–2020

Data regarding the CP funds for the period 2014–2020 were obtained from the EC data portal [40]. The data were extracted during 2017 for each one of the 385 operational programs (OP) of the current funding period (183 for ERDF, 155 for ESF, 22 for CF, and 25 ERDF–ESF) and included the following: (1) identifier; (2) title; (3) country code; (4) targeted region/city; (5) type of funding (ERDF, ESF, CF, or multi); (6) amount; (7) priority axes; (8) intervention codes.

From the 123 intervention field codes, organized in nine broader areas of interventions that represent the categories of actions or activities covered by the CP funds for the period 2014–2020, 59 codes were selected and used in the analyses (Table 2). In order to be included in the analysis, an intervention field code had to be linked to one or more population health dimensions of the PHI, meaning that the actions or activities described by the intervention field were directly or indirectly addressing one or more health determinants appraised within the PHI dimension.

### 2.2. Methods

Methods applied in this study aimed to explore the link between the current CP funding scheme and the dimensions of health determinants appraised in the EURO-HEALTHY PHI, looking to the regional performance in each of those dimensions.

In the first stage, several inferential statistics were applied to verify the presence of health inequalities across regions within each PHI dimension considering the regional level of development, i.e., the three categories for regional eligibility (LD, TR, and MD). In the first step, the Levene test for equality of variances was applied to verify the assumption of homogeneity of variance. Since the number of regions is different between categories and the PHI dimensions revealed unequal variance, the Welsh’s test was performed. Welch’s one-way analysis of variance (ANOVA) allowed for examining whether the different regional scores in each dimensional sub-index were statistically different across the three categories of regions. Then, the post hoc Games–Howell test was executed to identify which category mean was statistically different from the other two.

Finally, a two-dimensional matrix was built to represent the link between the CP categories for intervention and the PHI dimensions. Departing from this matrix and using the data on CP funds allocation by region and intervention field dimensions, descriptive statistics were used to identify those fields where the majority of funds (intervention fields with a higher number of NUTS 2) are concentrated and, in turn, verify the conformity with the priority intervention areas given by the PHI (dimensions with the lowest health scores by category of region).

## 3. Results

### 3.1. Population Health Index and Regional Level of Development

The ANOVA analysis revealed significant differences in the mean values of regional scores in most PHI dimensions (Table 3). In only three dimensions—education, aging, and road safety—regional scores did not vary significantly (*p*-value is not statistically significant).

**Figure 3:**
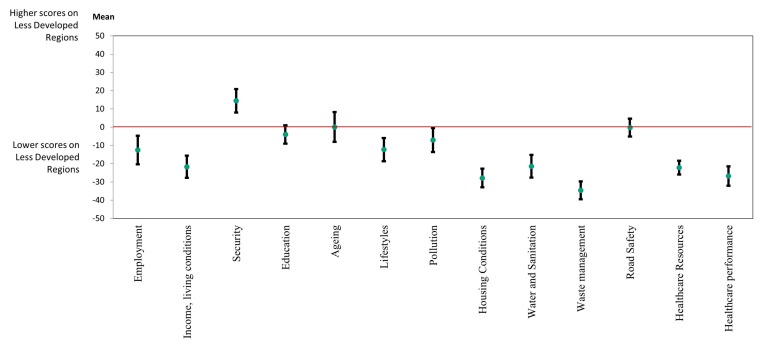
Mean differences between less developed (LD) regions and more developed (MD) regions, by dimensional sub-index. Note: The interval plots display 95% confidence intervals (CI) for each mean difference. Each dot represents the value of the mean difference in each dimensional sub-index: negative values in a dimension (below 0) indicate that LD regions perform worse than MD regions in that dimension; positive values in a dimension (above 0) indicate that LD regions perform better than MD regions in that dimension. If an interval does not contain 0, the corresponding means are significantly different.

Figure 3 presents the mean differences between LD and MD regions, by dimensional sub-index. In nearly all of the underlying health determinants included in the PHI, LD regions present worse scores when compared with MD regions. Scores presented by LD regions are particularly low in the dimensions of healthcare performance, housing conditions, and waste management, where the mean difference between the LD and MD regions was particularly higher (−27.8, −26.8, and −34.6, respectively).

### 3.2. Cohesion Policy Funds Allocation by Intervention Field and Category of Region

**Figure 4:**
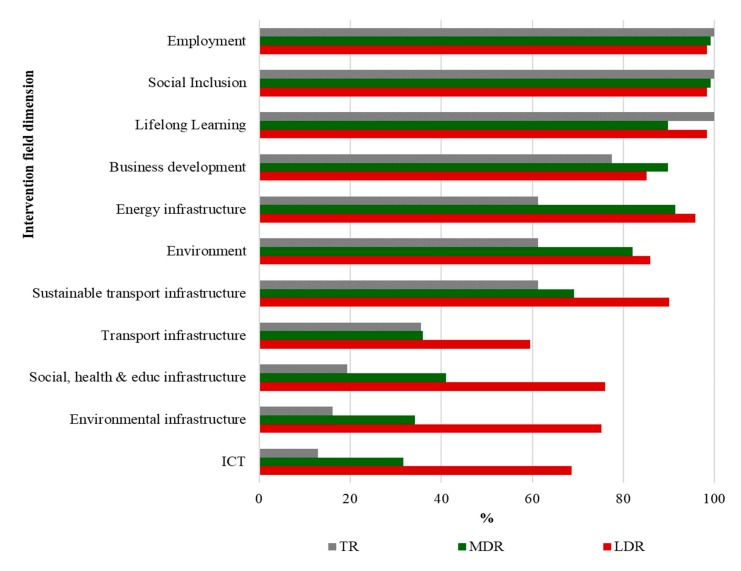
Percentage of NUTS 2 regions with allocated investment in each intervention field dimension, by category of region. Note: Numbers are calculated as a percentage of the number of regions within each category of region (LD, transition region (TR), and MD).

Figure 4 shows employment (98.9%), social inclusion (98.9%), and lifelong learning (94.8%) as the intervention field dimensions presenting the highest number of regions with planned investment. There is also a high proportion of regions allocating resources to energy infrastructure and to development of endogenous potential, specifically related to energy (90%) and business development (86.2%). LD regions are more prominently represented in the majority of the intervention fields, compared with TR and MD regions, particularly among the following fields: social, health and education infrastructure and related investment, environmental infrastructure, and endogenous potential: information and communications technology (ICT). For these three fields, substantially more programs and investments are planned amongst LD regions (73% on average) compared with TR and MD (16% and 35.6%, respectively on average). Supplementary Material 1 offers a series of maps depicting the geographical distribution of funds allocation by intervention field dimension across EU regions, highlighting LD regions.

The intervention field dimensions addressed most are (1) access to employment for job-seekers and inactive people, (2) energy efficiency renovation of public infrastructure, (3) active inclusion, (4) reducing and preventing early school leaving, and (5) adaptation of workers, enterprises and entrepreneurs. These interventions were the main priority investments for more than 90% of LD regions (see Supplementary Material 2). When looking at intervention fields addressing health directly, we can distinguish four types of actions: (1) enhancing access to affordable, sustainable, and high-quality health and social services (63.6%); (2) ICT solutions to assist with healthy active aging (60.3% of regions); (3) health infrastructure (54.5% of regions); (4) active and healthy aging (35.5%).

### 3.3. Cohesion Policy Categories of Intervention and the Population Health Index Dimensions

Table 4 presents the PHI dimensions, integrating the health determinants component, which are potentially addressed by the CP intervention fields defined for the period 2014–2020. Overall, 11 categories represent the intervention fields where actions address health determinants as measured by the PHI (see Table 1 to view the indicators within each dimension and Table 2 where information on the type of interventions within each intervention field dimension is provided).

The intervention field dimension inclusion (promoting social inclusion, combating poverty and discrimination) addresses almost all the PHI dimensions, with exceptions for pollution, water and sanitation, road safety, and healthcare performance. In contrast, there are three intervention fields that have impact only in one PHI dimension, such as (1) energy infrastructure and sustainable transport addressing pollution, and (2) lifelong learning addressing education. From the analysis of intervention fields, no interventions with impact on the dimension of healthcare performance were identified; this may well indicate the missing CP investment in that dimension. On the other hand, the PHI dimensions pollution, education, employment, and income and living conditions can be found in at least four CP intervention field dimensions. This means that these health determinants can be targeted through a set of CP fields.

Table 5 provides the number of regions within each category of region (LD, TR, and MD) that performed worse (scores below 50) in each dimension of the PHI, as well as how many from these regions indicated CP fund allocation in intervention fields addressing those dimensions.

Overall, half of the EU regions (135 out of 269) present low scores in one or more dimensions of health determinants; 97.5% are LD regions.

The dimensions of waste management and healthcare resources present the higher number of regions performing worse (respectively, 63 and 52 out of 269 regions); in more than one-third of LD regions (46 and 38 out of 121 LD regions), these dimensions are critical, presenting health scores below 50 (PHI ranges between 0 and 100). Conversely, road safety is the PHI dimension where only five regions show low health scores.

A correspondence exists between the dimensions identified as critical in each region and the respective CP allocated investment in intervention fields capable of improving indicators within those dimensions; this is the case for all dimensions, with the exception of aging, road safety, and healthcare performance, where there are regions that did not plan to allocate funds to any intervention field with potential impact on these dimensions. In the case of healthcare performance, no correspondence with the CP intervention fields was found.

## 4. Discussion

This paper aimed both to analyze the regional planned investments for allocating CP funds in the period 2014–2020 and to discuss their potential for reducing regional health inequalities in light of the evidence provided by the EURO-HEALTHY project and, specifically, by the population health index (PHI). The PHI collects data on key determinants of health in EU regions, providing insights into the factors which shape population health outcomes and fuel health inequalities. Its findings can have important implications for improving the process of setting priorities and of planning the allocation of CP funds which can be earmarked to positively impact health determinants.

Our first research question was to ascertain whether the intervention fields with planned allocation of CP funds were addressing the dimensions of health determinants where regions were lagging behind, that is, presenting worse scores in those dimensions.

Significant differences between the three categories of regions (LD, TR, and MD) were found in most PHI dimensions of health determinants. With the exception of pollution and security, LD regions present significantly worse population health scores in all dimensions of health determinants, compared with those presented by MD regions. There is a significant gap between the regions in the dimensions of housing conditions, waste management, and healthcare performance. Access to quality and affordable housing is a basic need for healthy living and is a key determinant measure to alleviate poverty and social exclusion [7]. CP funds can play an important role to ensure that every EU citizen, regardless the country or region of residence, has access to safe housing conditions. The group of regions that fall behind in terms of providing safe housing are often the new Member States (e.g., Bulgaria, Romania) [4]. CP investments addressing improved quality in housing, namely, the availability of sufficient living space within the dwelling, the existence of an indoor flushing toilet, and central heating, would contribute positively to obtaining better health scores in these regions. Central heating represents a core concern for population health in southern European regions [41] namely, Portugal, Spain, Italy, and Greece, where most of the population lives in homes without central heating (regional rates above 50%) [4].

On the other hand, regions across the EU, regardless of their level of development, face low health scores in the dimension of aging; no significant differences between LD and MD regions were found, meaning that all countries are experiencing an increase in their older populations. Nonetheless, unbalanced demographic structures could have a worse impact on fragile and less prepared social and health systems [35,42]. According to the literature, CP fund allocation can ameliorate those impacts though investment in categories of intervention dealing with, for example, “ICT solutions for healthy and active aging” [43], “lifelong learning opportunities” [44], “employment at an older age” [45], and “access to affordable, sustainable, and high-quality health and social services” [46].

The CP budget creates favorable conditions to achieve integrated development in population health-related fields, with guidelines that deal with how CP investments are directly applied into health systems to improve their effectiveness, accessibility, and resilience. However, the emphasis of CP on economic growth overshadows the importance of improving population health and overall well-being.

In general, the regional planned investments for CP funds are linked to the dimensions analyzed in the EURO-HEALTHY PHI. Most regions planned to allocate their funds to those interventions which represent a variety of multidimensional and multi-sectorial aspects influencing the health and well-being of people, as reflected in the rationale of the PHI.

Firstly, the most frequent correspondence was found between the PHI dimensions reflecting economic and living conditions and interventions that stimulate employment, support the labor market, promote social inclusion, and combat poverty. A high concentration of CP planned investments across regions are addressing the aforementioned domains, which the PHI identified as key dimensions responsible for polarization or social inequity across EU regions. Nearly all regions (95.9% and 86.6%) indicate the intention to allocate funds aimed at promoting active inclusion (with a view to promoting equal opportunities, active participation, and employability) and at enhancing equal access to lifelong learning for all ages. In 2015, the number of EU citizens at risk for poverty or social exclusion was estimated, on average, at 24.5% of the population, with countries such as Bulgaria, Greece, Hungary, Latvia, Lithuania, Romania, Portugal, Cyprus, and Croatia registering rates above the EU average. The highest rates of poverty and social exclusion were reported in the following LD regions: Sicilia, Italy (55.8%), Sud-Est, Romania (52.9%), and Campania, Italy (49%) [4].

Evidence from the PHI also confirms that large differences in education still exist across Europe: regions in north–central Europe present significantly higher health scores linked to good educational levels when compared with southern European regions (from Portugal, Spain, and Italy), where there is a concentration of worse performances. Dropping out of school is a multi-faceted and complex problem that increases the risk of unemployment, poverty, and social exclusion [47,48]. Another essential aspect addressed by CP funds pertains to education and combating school dropouts. The intervention fields of “reducing and preventing early school leaving” and “promoting equal access to good-quality early-childhood, primary, and secondary education” has planned investment in 90.1% of LD regions.

Secondly, aspects related with physical and built environment bring together a significant number of interventions addressing pollution, as they are related with energy efficiency, sustainable transport, and overall environmental infrastructures. Dimensions such as water and sanitation, waste management, and road safety are also addressed by a fair number of interventions. The research findings provide evidence that can help to confirm that CP can have a major impact on, for example, improving regional systems of waste collection and treatment [49,50,51]. A category of intervention with a significant amount of planned CP investment is “energy infrastructure”. Of reference are LD regions that are dedicating CP funds to “energy efficiency renovation of public infrastructure” (94.2% of regions), “energy efficiency renovation of existing housing stock” (88.4% of regions) and “clean urban transport infrastructure” (82.6% of regions), and “air quality measures” (62.8%). These interventions provide the opportunity to address the problems of high concentrations of air pollution (linked to intra-regional sources) among the LD regions (mostly east–central Europe), compared with their more affluent counterparts [4]. According to EURO-HEALTHY PHI, it is estimated that 157 million Europeans live in regions of significant concern in terms of the quality of one’s physical environment, measured by fine particulate matter (PM_2.5_), PM_10_, and greenhouse gas indicators, and in terms of receiving the lowest population health value score (physical environment index <50). These regions are mainly concentrated in east–central Europe [4] and are included in the group of countries allocating CP funds to interventions with potential to change the current air quality performance. Of mention are the priorities of investment in “energy infrastructure” and “sustainable transport infrastructure” (see Supplementary Material 2).

The second research question that this study aimed to explore was the verification of whether there were any categories and intervention fields that were identified as relevant for closing the regional gaps (considering the evidence of the PHI) but which appeared to be currently underrepresented in the CP planned investments. Results show that, while major upstream determinants of health seemed to be frequently addressed (e.g., employment, income and living conditions, education), other dimensions directly linked with the health sector remain underrepresented in funds allocation schemes. This is the case for healthcare performance, a dimension where a considerable number of regions are lagging behind, mostly LD regions from the Baltic and Eastern European countries (Latvia, Lithuania, Romania, Bulgaria, Slovakia, and Hungary). Worse scores in these regions call for urgent interventions aiming at both reducing amenable mortality due to healthcare and preventing hospital stays due to diabetes, hypertension, and asthma [4].

The current Third Health Program (2014–2020) seeks to promote innovative, efficient, and sustainable health systems, with EC encouraging MS to invest in the cost-effectiveness and sustainability of health systems and access to health services for all social groups [52]. Although opportunities for health investments through European Structural and Investments Funds (ESIF) (including CP funds) were widely communicated by the Commission [16,53], this area remains rather unexploited. Fund allocation can be significantly improved, given that the health sector in Europe accounts for 10% of the EU’s GDP and represents the core of the EU’s social protection [52].

Given its ability to provide a comprehensive picture of big data through indices, the PHI presents the potential to navigate fund allocation in a direction of greater health equity across European regions. This would also allow for the necessary expanding of those eligibility criteria that are currently narrowed to GDP per capita, despite the common evidence that this is only one indicator amongst many. The Juncker Plan for improved and more strategic use of CP and ESIF (also in combination) is already incorporating some of these needs. Moreover, the EC’s proposal for a future strategy for the 2020–2027 budget period suggests new criteria, in addition to GDP, for analyzing fund allocation. As an assessment tool, the PHI will allow for improved target specific fund allocation to local and regional specificities and needs of EU regions.

The PHI could act as an informing tool that could facilitate a more focused and targeted distribution of CP funds. This rationale is also in line with the 2018 report of the multi-stakeholder platform on the implementation of the United Nations sustainable development goals (SDGs) in the EU, which seeks to advise the EC on implementing the SDGs through the adjustment of the next multi-annual financial framework. By underscoring the importance of the 2030 Agenda for Sustainable Development, which has at its core the 17 SDGs with 169 targets, the report suggests the need to restructure the CP funds for the post-2020 period, specifically by adjusting the allocation criteria through new indicators to better reflect economic, social, and environmental aspects. The argument presented is that it is counterproductive to develop funding frameworks which incentivize expenditure that is harmful to the environment and health or likely to increase disparities in well-being. The determination as to which regions will receive CP funding should be supported by a much more refined basis of evidence that not only identifies those regions which lag behind, but also makes reference to specific dimensions and indicators. The PHI, through its model disaggregated into dimensions and indicators, can provide useful information for the potential establishment of a new scheme of funding which goes beyond the GDP as the only measure on which the definition of regional eligibility categories is based.

### Limitations

CP funds are allocated in ways that indirectly consider health determinants and health outcomes. Further research should be conducted to identify the types of evidence and instruments that are required to support the EU and MS efforts to ensure that funds are most effectively and efficiently allocated to regions with the highest need.

For example, it would be highly relevant to study the implementation effectiveness of the use of CP funds addressing the PHI dimensions of health determinants considering its potential benefit to reduce the regional gaps in health. Moreover, the analysis of the previous (2007–2013) and current programming periods (2014–2020), upon conclusion of the latter, will enable comparisons to be drawn that may support a better allocation of funding in the future programming period.

The analysis of the OPs focuses solely on the three fund categories: ERDF, ESF, and CF. There are additional funds available within the ESIF that have the potential to support the mitigation of health inequalities across European regions, mainly the Agricultural Fund for Rural Development (EAFRD), the European Maritime and Fisheries Fund (EMFF), and European Territorial Cooperation (ETC): Interreg V. Nevertheless, they were beyond the scope of the present study’s analysis.

Although the ANOVA analysis demonstrated that, in the majority of PHI dimensions, the regional population health scores present significant statistical variations by category (LD, TR, and MD), some caution is needed when analyzing the results. The independence of the observations (in this study, the regions) is one of the main assumptions of ANOVA. We defend the independence of the data regarding the population health index because the value score of one region did not depend on the value score of another region. Yet, we could not establish independence regarding the regional eligibility categories since they are not randomly assigned (regions are categorized by the EC according to their respective level of development as measured by GDP) [20].

Moreover, data from the PHI used in this study cover year 2014, and specific data gaps were identified throughout the EU regions and PHI indicators. Constraints on data availability at the regional level were found in dimensions within built environment, lifestyle and health behaviors, and healthcare performance [39].

Lastly, it is acknowledged that there are a variety of considerations which come into play during the process of setting funding priorities. One is the wide variation in the design, management, and performance of health systems across the EU, which may impact the level of supply and demand of healthcare in each country. Another may be the different forms of governance and the administrative capacity of national and regional authorities, which represent different levels of bureaucracy and may in turn influence the allocation of funds amongst competing priorities and planned investments.

Given these considerations, the PHI can provide additional evidence to apply in these negotiations and decision-making processes, promoting an evidence-based approach which the EC, as well as the MS and regional governments themselves, can use to develop guidelines for funding.

## 5. Conclusions

This study adds evidence to the current need to rethink how appropriately a country’s GDP may serve as the sole eligibility criterion for allocating regional funds. The level of development measured by GDP is not a direct indication that a region with high GDP has good levels of health and well-being because population health is multidimensional and includes many factors beyond the level of economic development and income. Results shown by the EURO-HEALTHY PHI not only demonstrate that key dimensions of population health equity are underrepresented in the current funding scheme but also that regions with high GDP (classified as MD regions) present worse scores in important determinants negatively affecting health and well-being, such as public safety (a dimension of security) and air quality (a dimension of pollution).

The dimensions of aging, road safety, and healthcare performance appear to be currently underrepresented in CP planned investment. In fact, in the case of healthcare performance, no correspondence with the CP intervention fields was found. This shows that there is room of improvement on how to use cohesion funds to improve the health systems’ effectiveness, accessibility and resilience, and accordingly achieve an integrated development in population health-related fields.

A fresh approach is needed to extend the current eligibility criteria into more dimensions. Using GDP as the sole measurement of eligibility presents limitations when considering the factors shaping the regional gaps across Europe. It is well known that GDP fails as a measure of well-being and health. Given that health is multidimensional and the effective way to promote health and that reducing inequities in health means acting on their causes (social, economic, and environmental determinants), it becomes extremely important to involve all policy sectors and entities acting on these determinants. A more intersectoral and integrated approach—Health in All Policies—is needed in all phases, from planning the investments to their implementation.

## Figures and Tables

**Table 1:** Population health index (PHI) structure.

Area of Concern	Dimension	Indicators
**Health Determinant Index**		
Economic conditions, social protection, and security	Employment	Unemployment rate (%)
		Long-term unemployment rate—12 months and more (%)
	Income and living conditions	Disposable income of private households per capita (euros per inhabitant)
		People at risk of poverty or social exclusion (%)
		Disposable income ratio—S80/S20 (ratio)
	Social protection	Expenditure on care for the elderly (% of gross domestic product (GDP))
	Security	Crimes recorded by the police (per 100,000 inhabitants)
Education	Education	Population aged 25–64 with upper secondary or tertiary education attainment (%)
		Early leavers from education and training (%)
Demographic change	Aging	At risk of poverty rate of older people—aged 65 years and over (%)
		Aging index (ratio)
Lifestyle and health behaviors	Lifestyle and health behaviors	Adults who are obese (%)
		Daily smokers—aged 15 and over (%)
		Pure alcohol consumption—aged 15 and over (liters per capita)
		Live births by mothers under age of 20 (%)
Physical environment	Pollution	Annual mean of the daily fine particulate matter (PM_2.5_) concentrations (µg/m^3^)
		Annual mean of the daily PM_10_ concentrations (µg/m^3^)
		Greenhouse gas (total tons of CO_2_ equivalent emissions per capita)
		Population exposed to traffic noise—Lden55–59db, during day (%) *
	Extreme weather events *	Population affected by flooding (per 1,000,000 inhabitants) *
Built environment	Housing conditions	Average number of rooms per person
		Households without indoor flushing toilet (%)
		Households without central heating (%)
	Water and sanitation	Population connected to public water supply (%)
		Population connected to wastewater treatment plants (%)
	Waste Management	Recycling rate of municipal waste (%)
	Land use *	Population density (inhabitants/km^2^) *
Road safety	Road safety	Victims in road accidents—injured and killed (per 100,000 inhabitants)
		Fatality rate due to road traffic accidents (per 1000 victims)
Healthcare resources and expenditure	Healthcare resources	Medical doctors (per 100,000 inhabitants)
		Health personnel—nurses and midwives, dentists, pharmacists, and physiotherapists (per 100,000 inhabitants)
	Healthcare expenditure	Total health expenditure (purchasing power standard per capita)
		Private household out-of-pocket expenses on health (% of total health expenditure)
		Public expenditure on health ( purchasing power standard per capita)
Healthcare performance	Healthcare performance	Hospital discharges due to diabetes, hypertension, and asthma (per 100,000 inhabitants)
		Amenable deaths due to healthcare (standardized death rate per 100,000 inhabitants)
**Health Outcome Index**		
Health outcomes	Length of life (mortality)	Life expectancy at birth (years)
		Infant mortality (per 1000 live births)
		Preventable deaths (standardized death rate per 100,000 inhabitants)
	Quality of life(morbidity)	Self-perceived health less than good (%)
		Age-standardized disability-adjusted life year (DALY) rate (per 100,000 inhabitants)
		Low birth weight (%)

* Dimensions and indicators included in the PHI model (conceptual model) but not used in its application (adjusted model) to the 269 NUTS (Nomenclature of Territorial Units for Statistics) 2 regions, due to lack of data.

**Table 2:** Cohesion Policy funding categories and intervention fields for the period 2014–2020.

Categories and Intervention Field Dimension	Code	Intervention Field
A—Energy infrastructure	009	Renewable energy: wind
	010	Renewable energy: solar
	011	Renewable energy: biomass
	012	Other renewable energy (including hydroelectric, geothermal, and marine energy) and renewable energy integration (including storage, power to gas, and renewable hydrogen infrastructure)
	013	Energy efficiency renovation of public infrastructure, demonstration projects and supporting measures
	014	Energy efficiency renovation of existing housing stock, demonstration projects, and supporting measures
	015	Intelligent Energy Distribution Systems at medium and low voltage levels (including smart grids and information and communication technology (ICT) systems)
	016	High-efficiency co-generation and district heating
A—Environmental infrastructure	017	Household waste management (including minimization, sorting, recycling measures)
	018	Household waste management (including mechanical biological treatment, thermal treatment, incineration, and landfill measures)
	019	Commercial, industrial, or hazardous waste management
	020	Provision of water for human consumption (extraction, treatment, storage, and distribution infrastructure)
	021	Water management and drinking water conservation (including river basin management, water supply, specific climate change adaptation measures, district and consumer metering, charging systems, and leak reduction)
	022	Wastewater treatment
	023	Environmental measures aimed at reducing and/or avoiding greenhouse gas emissions (including treatment and storage of methane gas and composting)
A—Transport infrastructure	028	Trans-European Transport Network (TEN-T) motorways and roads—core network (new build)
	029	Trans-European Transport Network (TEN-T) motorways and roads—comprehensive network (new build)
	030	Secondary road links to TEN-T road network and nodes (new build)
	031	Other national and regional roads (new build)
	032	Local access roads (new build)
	033	Trans-European Transport Network (TEN-T) reconstructed or improved road
	034	Other reconstructed or improved road (motorway, national, regional, or local)
A—Sustainable transport	043	Clean urban transport infrastructure and promotion (including equipment and rolling stock)
	044	Intelligent transport systems (including the introduction of demand management, tolling systems, IT monitoring, control and information systems)
B—Public facilities	049	Education infrastructure for tertiary education
	050	Education infrastructure for vocational education and training and adult learning
	051	Education infrastructure for school education (primary and general secondary education)
	052	Infrastructure for early childhood education and care
	053	Health infrastructure
	054	Housing infrastructure
	055	Other social infrastructure contributing to regional and local development
C—Business development	068	Energy efficiency and demonstration projects in Small and Medium-sized Enterprises (SMEs) and supporting measures
	069	Support to environmentally friendly production processes and resource efficiency in SMEs
	070	Promotion of energy efficiency in large enterprises
	071	Development and promotion of enterprises specialized in providing services contributing to the low-carbon economy and to resilience to climate change (including support to such services)
C—ICT	080	E-inclusion, e-accessibility, e-learning, and e-education services and applications, digital literacy
	081	ICT solutions addressing the healthy active aging challenge, and e-health services and applications (including e-care and ambient assisted living)
C—Environment	083	Air quality measures
	084	Integrated pollution prevention and control (IPPC)
	085	Protection and enhancement of biodiversity, nature protection, and green infrastructure
	089	Rehabilitation of industrial sites and contaminated land
	090	Cycle tracks and footpaths
D—Employment	102	Access to employment for jobseekers and inactive people, including the long-term unemployed and people far from the labor market, also through local employment initiatives and support for labor mobility
	103	Sustainable integration into the labor market of young people, those not in employment, education or training, including young people at risk of social exclusion and young people from marginalized communities, including through the implementation of the youth guarantee
	104	Self-employment, entrepreneurship and business creation including innovative micro, small- and medium-sized enterprises
	105	Equality between men and women in all areas, including in access to employment, career progression, reconciliation of work and private life, and promotion of equal pay for equal work
	106	Adaptation of workers, enterprises, and entrepreneurs to change
	107	Active and healthy aging
	108	Modernization of labor market institutions, such as public and private employment services, and improving the matching of labor market needs, including through actions that enhance transnational labor mobility, as well as through mobility schemes and better cooperation between institutions and relevant stakeholders
E—Inclusion	109	Active inclusion, including with a view to promoting equal opportunities and active participation, and improving employability
	110	Socio-economic integration of marginalized communities such as Roma
	111	Combating all forms of discrimination and promoting equal opportunities
	112	Enhancing access to affordable, sustainable, and high-quality services, including healthcare and social services of general interest
	113	Promoting social entrepreneurship and vocational integration in social enterprises and the social and solidarity economy in order to facilitate access to employment
	114	Community-led local development strategies
F—Lifelong learning	115	Reducing and preventing early school-leaving and promoting equal access to good-quality early-childhood, primary, and secondary education including formal, non-formal, and informal learning pathways for reintegrating into education and training
	116	Improving the quality and efficiency of, and access to, tertiary and equivalent education with a view to increasing participation and attainment levels, especially for disadvantaged groups
	117	Enhancing equal access to lifelong learning for all age groups in formal, non-formal, and informal settings, upgrading the knowledge, skills, and competences of the workforce, and promoting flexible learning pathways including through career guidance and validation of acquired competences
	118	Improving the labor market relevance of education and training systems, facilitating the transition from education to work, and strengthening vocational education and training systems and their quality, including through mechanisms for skills anticipation, adaptation of curricula, and the establishment and development of work-based learning systems, including dual learning systems and apprenticeship schemes

Source: Commission Implementing Regulation (EU) No 215/2014, of 7 March 2014, Annex I—Nomenclature for the categories of intervention of the Funds under the Investment for growth and jobs goal and of the Youth Employment Initiative. Main categories of intervention (European Regional Development Fund (ERDF), the European Social Fund (ESF), and the Cohesion Fund (CF)): A—infrastructure providing basic services and related investment; B—social, health, and education infrastructure investment; C—development of endogenous potential; D—promoting sustainable and quality employment and labor mobility; E—promoting social inclusion, combating poverty, and any discrimination; F—investing in education, training, and vocational training for skills and lifelong learning.

**Table 3:** ANOVA statistics by dimensional sub-index.

Population Health Index Dimensions—Health Determinants	Welch’s *t*-Test	*p*-Value
Employment	15.112	0.000 *
Income and living conditions	32.879	0.000 *
Security	22.979	0.000 *
Education	7.488	0.001
Aging	0.899	0.410
Lifestyles and health behaviors	28.000	0.000 *
Pollution	11.899	0.000 *
Housing conditions	57.107	0.000 *
Water and sanitation	27.124	0.000 *
Waste management	79.516	0.000 *
Road safety	0,186	0.831
Healthcare resources	82.760	0.000 *
Healthcare performance	38.083	0.000 *

Note: * *p*-value < 0.001.

**Table 4:** Matrix of Cohesion Policy intervention field dimensions and the EURO-HEALTHY population health index dimensions.

	Population Health Index Dimensions—Health Determinants												
Intervention Field Dimensions	Employment	Income, Living Conditions	Security	Education	Aging	Lifestyles	Pollution	Housing Conditions	Water and Sanitation	Waste Management	Road Safety	Healthcare Resources	Healthcare Performance
Energy infrastructure							•						
Environmental infrastructure							•		•	•			
Transport infrastructure											•		
Sustainable transport							•						
Public facilities		•		•				•				•	
Business development	•						•						
ICT	•	•		•	•							•	
Environment						•	•						
Employment	•	•			•								
Inclusion	•	•	•	•	•	•		•		•		•	
Lifelong learning				•									

Note: The dots indicate a relationship between the intervention field dimension and the PHI dimensional health sub-index (e.g., interventions made in energy infrastructure have a potential impact on the indicators that integrate the dimension of pollution).

**Table 5:** Number of European Union (EU) regions with the lowest scores and with CP allocated investment, by category of region and dimensional health sub-index.

PHI Dimensional Health Sub-Indices	Number of Regions with Scores Below 50				Number of Regions with the Lowest Scores and with Allocated Investment			
	LD	TR	MD	Total	LD	TR	MD	Total
Employment	10	10	2	22	10	10	2	22
Income and living conditions	17	4	1	22	17	4	1	22
Security	5	12	14	31	5	12	14	31
Education	8	7	4	19	8	7	4	19
Aging	9	7	5	21	9	7	4	20
Lifestyles and health behaviors	14	0	0	14	14	-	-	14
Pollution	14	1	22	37	14	1	22	37
Housing conditions	13	0	0	13	13	-	-	13
Water and sanitation	10	0	2	12	10	-	2	12
Waste management	46	8	9	63	46	8	9	63
Road safety	2	0	3	5	2	-	0	2
Healthcare resources	38	8	6	52	38	8	6	52
Healthcare performance	24	0	3	27	0	-	0	0

Note: Calculations based on the matrix of CP intervention field dimensions (see Table 4) and the EURO-HEALTHY PHI dimensions (see Table 1). For each region, dimensions presenting value scores below 50 (PHI ranges between 0 and 100) and CP intervention field dimensions with planned investment were identified. For a region to be considered one with allocated investment addressing the PHI dimension, it should present at least one CP intervention field dimension with potential impact on the dimensional health sub-index with lowest scores.

## Supplementary Materials

The following are available online at https://www.mdpi.com/1660-4601/17/5/1567/s1. Supplementary Material 1: CP fund allocation by intervention field dimensions and category of region. Figure S1: Allocation of funds by category of intervention across NUTS 2 level regions, 2014–2020. Supplementary Material 2: Less developed regions: priorities of investment and population health dimension addressed; Table S2: CP intervention fields by number of LD regions with planned investment and respective PHI dimensions addressed.
