# Supplementary material for: The Role of Cohesion Policy Funds in Decreasing the Health Gaps Measured by the EURO-HEALTHY Population Health Index
Source: Int J Environ Res Public Health. 2020 Feb 29;17(5):1567. doi: 10.3390/ijerph17051567 (PMC7084523; doi:10.3390/ijerph17051567)
Supplement: Supplementary file 1 [file ijerph-17-01567-s001.zip › Supplementary material/S1.docx]

**Supplementary material 1: CP funds allocation by intervention field dimensions and category of region**


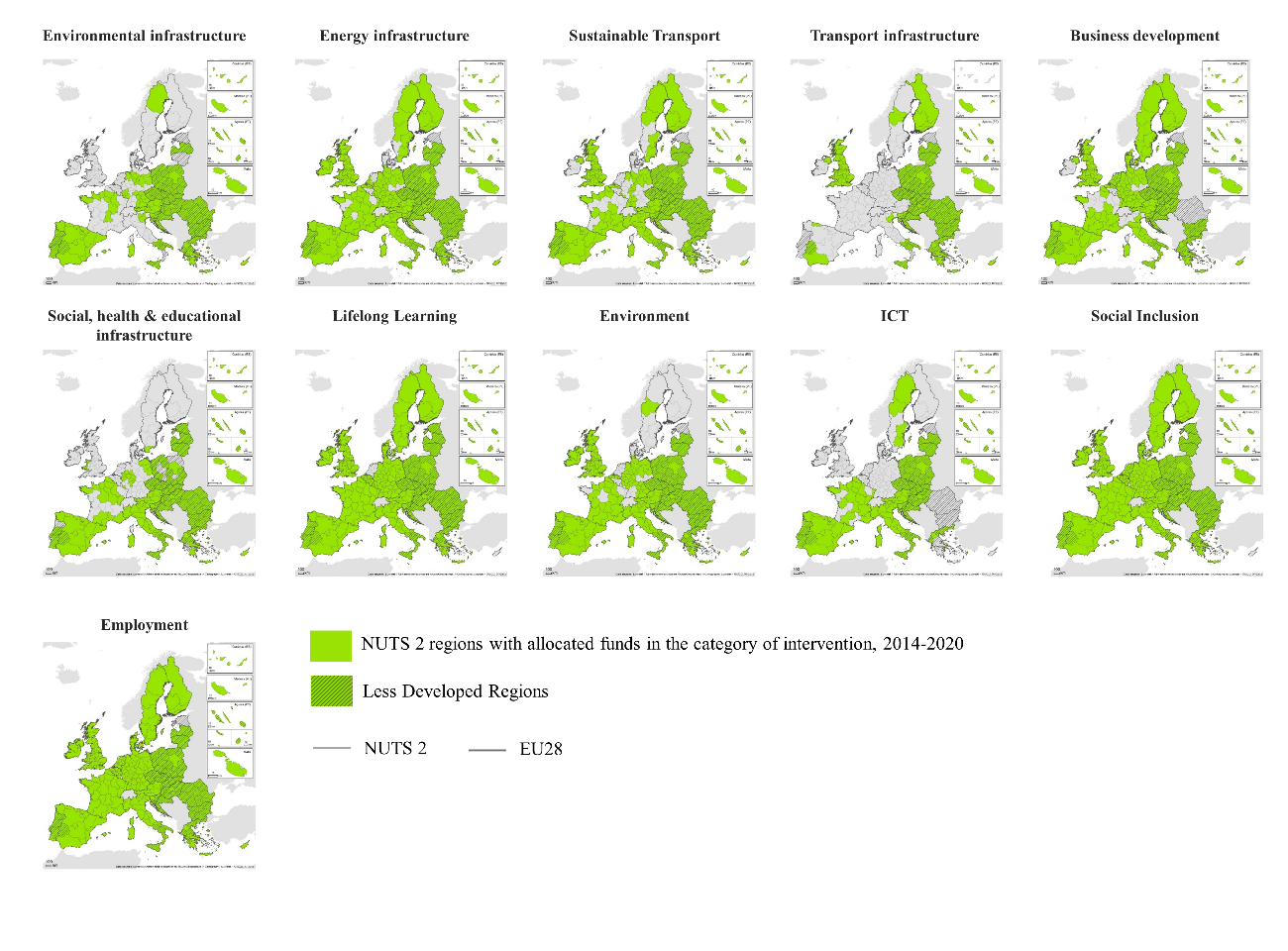


Figure S1. Allocation of funds by category of intervention across NUTS 2 level regions, 2014-2020
