# Supplementary material for: The Role of Cohesion Policy Funds in Decreasing the Health Gaps Measured by the EURO-HEALTHY Population Health Index
Source: Int J Environ Res Public Health. 2020 Feb 29;17(5):1567. doi: 10.3390/ijerph17051567 (PMC7084523; doi:10.3390/ijerph17051567)
Supplement: Supplementary file 1 [file ijerph-17-01567-s001.zip › Supplementary material/S2.docx]

**Supplementary material 2. Priorities of investment among the LD regions and population health dimension addressed**

Table S2. CP intervention fields by number of LD regions with allocated investment and respective PHI dimensions addressed

| **Intervention field dimension** | **Intervention field** | **EURO-HEALTHY PHI Dimension (potentially) addressed** | **LD (N=121) %** |
| --- | --- | --- | --- |
| Employment | Access to employment for job-seekers and inactive people | Employment; Income & living conditions | 97.5 |
| Energy infrastructure | Energy efficiency renovation of public infrastructure | Pollution | 94.2 |
| Social Inclusion | Active inclusion | Employment | 92.6 |
| Employment | Adaptation of workers, enterprises and entrepreneurs to change | Employment | 90.1 |
| Lifelong Learning | Reducing and preventing early school-leaving | Education | 90.1 |
| Energy infrastructure | Energy efficiency renovation of existing housing stock | Pollution | 88.4 |
| Sustainable transport infrastructure | Clean urban transport infrastructure and promotion | Pollution | 82.6 |
| Energy infrastructure | Renewable energy: biomass | Pollution | 81.8 |
| Energy infrastructure | Other renewable energy (hydroelectric, geothermal, marine energy) | Pollution | 81.0 |
| Lifelong Learning | Enhancing equal access to lifelong learning for all age groups | Employment; Education | 81.0 |
| Employment | Modernisation of labour market institutions | Employment | 80.2 |
| Employment | Sustainable integration into the labour market of young people | Employment; Income & living conditions | 80.2 |
| Sustainable transport infrastructure | Intelligent transport systems | Pollution | 77.7 |
| Lifelong Learning | Improving the quality and efficiency of, and access to, tertiary education | Education | 76.9 |
| Environment | Rehabilitation of industrial sites and contaminated land | Pollution | 76.0 |
| Environment | Protection of biodiversity, nature protection and green infrastructure | Pollution | 75.2 |
| Social, health & educational infrastructure | Other social infrastructure contributing to regional and local development | Income & Living conditions | 70.2 |
| Employment | Self-employment, entrepreneurship and business creation | Employment; Income & living conditions | 68.6 |
| Environment | Cycle tracks and footpaths | Lifestyles; Pollution | 68.6 |
| Environmental infrastructure | Waste water treatment | Water and sanitation | 68.6 |
| Lifelong Learning | Improving the labour market relevance of education and training systems | Employment; Education | 66.1 |
| Business development | Energy efficiency and demonstration projects in SMEs | Pollution | 63.6 |
| Social Inclusion | Enhancing access to affordable, sustainable and high-quality health & social services | Income & living conditions | 63.6 |
| Employment | Equality between men and women in all areas | Employment; Income & living conditions | 62.8 |
| Environment | Air quality measures | Pollution | 62.8 |
| Business development | Support to environmentally-friendly processes and resource efficiency | Pollution | 61.2 |
| ICT | ICT solutions for healthy active ageing and e-Health applications | Employment; Ageing; Healthcare resources | 60.3 |
| ICT | e-Inclusion, e-Accessibility, e-Learning and e-Education services | Employment; Income & Living conditions, Education; Healthcare resources | 60.3 |
| Environmental infrastructure | Water management and drinking water conservation | Water and sanitation | 59.5 |
| Social Inclusion | Promoting social entrepreneurship and vocational integration in social enterprises | Employment | 59.5 |
| Social Inclusion | Socio-economic integration of marginalised communities such as the Roma | Income & living conditions | 59.5 |
| Environmental infrastructure | Household waste management (recycling) | Waste management | 57.9 |
| Energy infrastructure | Renewable energy: solar | Pollution | 57.0 |
| Environmental infrastructure | Household waste management (treatment) | Waste management | 56.2 |
| Transport infrastructure | Other reconstructed or improved road (national, regional or local) | Road safety | 55.4 |
| Social, health & educational infrastructure | Health infrastructure | Healthcare resources | 54.5 |
| Energy infrastructure | Intelligent Energy Distribution Systems | Pollution | 53.7 |
| Social, health & educational  infrastructure | Education infrastructure for school education | Education | 52.1 |
| Environmental infrastructure | Provision of water for human consumption | Water and sanitation | 50.4 |
| Social, health & educational infrastructure | Housing infrastructure | Housing conditions | 50.4 |
| Energy infrastructure | High efficiency co-generation and district heating | Pollution | 49.6 |
| Social, health & educational infrastructure | Education infrastructure for tertiary education | Education | 47.1 |
| Social Inclusion | Combating all forms of discrimination and promoting equal opportunities | Employment; Income & living conditions; Education; Ageing; Housing conditions | 46.3 |
| Social, health & educational infrastructure | Infrastructure for early childhood education and care | Education | 45.5 |
| Social, health & educational infrastructure | Education infrastructure for vocational education and training and adult learning | Education | 45.5 |
| Social Inclusion | Community-led local development strategies | Employment; Income & living conditions; Security; Education; Ageing; Lifestyles; Housing conditions; Waste management; Healthcare resources | 41.3 |
| Energy infrastructure | Renewable energy: wind | Pollution | 36.4 |
| Employment | Active and healthy ageing | Ageing | 35.5 |
| Transport infrastructure | Secondary road links to TEN-T road network and nodes (new build) | Road safety | 33.9 |
| Business development | Development and promotion of enterprises of low carbon economy | Employment; Pollution | 33.1 |
| Environmental infrastructure | Commercial, industrial or hazardous waste management | Waste management | 32.2 |
| Transport infrastructure | TEN-T reconstructed or improved road | Road safety | 32.2 |
| Business development | Promotion of energy efficiency in large enterprises | Pollution | 28.9 |
| Environmental infrastructure | Environmental measures aimed at reducing GHG emmissions | Pollution | 28.9 |
| Transport infrastructure | TEN-T motorways and roads - comprehensive network (new build) | Road safety | 28.9 |
| Transport infrastructure | TEN-T motorways and roads network (new build) | Road safety | 25.6 |
| Transport infrastructure | Other national and regional roads (new build) | Road safety | 25.6 |
| Transport infrastructure | Local access roads (new build) | Road safety | 18.2 |
| Environment | Integrated pollution prevention and control (IPPC) | Pollution | 14.0 |

Source: Authors' calculations on the EC data portal (<https://cohesiondata.ec.europa.eu>).

Note: the number of regions was calculated as a percentage of the number of regions classified as LDR with allocated funding in each intervention field code.

LD – Less Developed Regions
